# A Web-Based Tool to Perform a Values Clarification for Stroke Prevention in Patients With Atrial Fibrillation: Design and Preliminary Testing Study

**DOI:** 10.2196/67956

**Published:** 2025-04-11

**Authors:** Michael P Dorsch, Allen J Flynn, Kaitlyn M Greer, Sabah Ganai, Geoffrey D Barnes, Brian Zikmund-Fisher

**Affiliations:** 1 College of Pharmacy, University of Michigan, 248 Church Street, Ann Arbor, MI, 48109, United States, 17347647312; 2 Department of Learning Health Sciences, Medical School, University of Michigan, Ann Arbor, MI, United States; 3 Division of Cardiovascular Medicine, Medical School, University of Michigan, Ann Arbor, MI, United States; 4 School of Public Health, University of Michigan, Ann Arbor, MI, United States

**Keywords:** digital health, atrial fibrillation, stroke prevention, shared decision-making, values clarification

## Abstract

**Background:**

Atrial fibrillation (AF) is associated with an increased risk of stroke. Oral anticoagulation (OAC) is used for stroke prevention in AF, but it also increases bleeding risk. Clinical guidelines do not definitively recommend for or against OAC for patients with borderline stroke risk. Decision-making may benefit from values clarification exercises to communicate risk trade-offs.

**Objective:**

This study aimed to evaluate if a visual with a values clarification alters the understanding of the trade-offs of anticoagulation in AF.

**Methods:**

Participants aged 45‐64 years were recruited across the United States via an online survey. While answering the survey, they were asked to imagine they were newly diagnosed with AF with a CHA_2_DS_2_-VASc (congestive heart failure; hypertension; age ≥75 years [doubled]; type 2 diabetes; previous stroke, transient ischemic attack, or thromboembolism [doubled]; vascular disease; age 65 to 75 years; and sex category) score of 1 for men and 2 for women. Eligibility criteria included no diagnosis of AF and no prior OAC use. Participants were randomized to one of three conditions: (1) standard text-based information only (n=255), (2) visual aids showing stroke-risk probabilities (n=218), or (3) visual aids plus a values clarification exercise (visual+VC; n=200). Participants were subrandomized within the 2 visual-based groups to view either a gauge display or an icon array representing stroke risk. All participants read a hypothetical scenario of being newly diagnosed with AF and hypertension. The primary outcome was decision confidence as measured by the SURE (Sure of Myself; Understand Information; Risk-Benefit Ratio; Encouragement) test. Secondary measures included participants’ perceived stroke risk reduction, worry about stroke or bleeding, and likelihood to choose OAC.

**Results:**

A total of 673 participants completed the survey. The overall SURE test was 61.2% (156/255) for the standard, 66.5% (145/218) for the visual, and 67% (134/200) for the visual+VC group (visual vs standard *P*=.23; visual+VC vs standard *P*=.20). Participants were less likely to choose OAC in the visual groups (standard: mean 58.3, SD 30; visual: mean 51.4, SD 32; visual+VC: 51.9, SD 28; *P*=.03). Participants felt the reduction in stroke risk from an OAC was less in the visual groups (standard: mean 63.8, SD 22; visual: mean 54.2, SD 28; visual+VC: mean 58.6, SD 25; *P*<.001). Visualization methods (gauge vs icon array) showed no significant differences in overall SURE test results. Participants were less likely to choose OAC and perceived a smaller stroke risk reduction with gauge than icon array (OAC choice: gauge 48.8, icon array 55.4; *P*=.03; stroke risk reduction: gauge 52.1, icon array 60.4; *P*=.001).

**Conclusions:**

Visual aids can modestly affect decision confidence and perceptions regarding the benefits of OAC but do not significantly alter decision certainty in a scenario where the guidelines do not recommend for or against OAC. Future work should determine the role of a gauge versus icon array visual for decision-making in stroke prevention in AF.

## Introduction

Risk stratification and shared decision-making are essential in stroke prevention in atrial fibrillation (SPAF). In a wide variety of patients with AF, anticoagulation reduces the risk of ischemic stroke by 65% with a relative 2-fold increase in major extracranial bleeding compared to placebo [1-3]. Yet, medication responses vary across patients. Personalized risks and benefits are available to clinicians via the CHA_2_DS_2_-VASc (congestive heart failure; hypertension; age ≥75 years [doubled]; type 2 diabetes; previous stroke, transient ischemic attack, or thromboembolism [doubled]; vascular disease; age 65 to 75 years; and sex category) and HAS-BLED (hypertension, abnormal renal/liver function, stroke, bleeding history or predisposition, labile international normalized ratio, elderly [>65 years], drugs/alcohol concomitantly) risk scoring systems, representing the risk of stroke and bleeding in AF [4-6]. These tools can provide a tailored estimate of a patient’s benefit and risk of anticoagulation in AF.

Many current AF-shared decision-making tools use visual tools such as icon arrays to display the percent risk of stroke (CHA_2_DS_2_-VASc) and risk of bleed (HAS-BLED). While such tools help convey probabilities to patients [7], such probability-focused communications do not visually distinguish between different outcomes. This is a problem because it may lead patients and clinicians to give similar weight to these outcomes even though the medical complications of a stroke are far greater than the medical complications of a bleed. AF guidelines indicate that for the majority of patients where anticoagulation is recommended (CHA_2_DS_2_-VASc ≥2), the HAS-BLED is best used to remove or treat risk factors for bleeding (eg, stop concomitant aspirin or nonsteroidal anti-inflammatory drugs and treat hypertension) rather than to determine if anticoagulation should or should not be given.

One approach to encouraging more thoughtful consideration of the different possible outcomes of AF is using values clarification exercises [3]. Values clarification exercises are structured activities that encourage people to consider how much subjective weight they place on different possible outcomes [8-10]. For many years, developers of patient decision aids have encouraged the inclusion of values clarification exercises in such tools to increase the alignment of medical decisions with patient preferences. However, there is limited evidence on the comparative effectiveness of these different formats in the context of oral anticoagulation (OAC) decision-making in AF.

We report the results of a multistep design and evaluation process to explore the potential for integrating values clarification exercise–derived patient values into presentations of the risks and benefits of anticoagulant therapy. We based our work on the Ottawa Decision Support Framework (ODSF), an evidence-based midrange theory guiding patients’ health decisions [11,12]. The framework is based on concepts from psychology, decision analysis, and decision conflict to evaluate the quality of outcomes in providing decision support. In this project, we engaged patients and providers in the user-centered design of a decision support tool for anticoagulation in AF (ODSF step 1), built the technology to deliver this tailored decision support tool (ODSF step 2), and tested if the decision support tool with a values clarification improves the knowledge of the trade-offs of anticoagulation in AF (ODSF step 3).

## Methods

### Study Design

We used a user-centered design to develop the decision support tool. For the user-centered design, we conducted an iterative series of user experience interviews with adults recruited from the general population, medical providers, and patient-provider dyads. We recruited participants from the general Ann Arbor, Michigan, population participants during February or March 2020 (first round), April 2020 (second round), and May 2020 (third round). In addition to these general patient interviews, we interviewed 6 providers and performed 2 patient-provider dyad interviews. These patient interviews were conducted virtually due to the COVID-19 pandemic.

After completing the design of the decision support tool, we performed a randomized controlled trial using a sample of adults recruited from across the United States using a panel managed by the online survey company Qualtrics. Participants were eligible if they were 45 to 64 years old, had not been diagnosed with AF, and had not taken anticoagulants.

The Qualtrics-administered survey asked participants to imagine themselves as a patient diagnosed with AF and hypertension, which made the imaginary patient a CHA_2_DS_2_-VASc score of 1 for men and 2 for women. This was chosen because using anticoagulation in those patients is not definitive in the guidelines, and patients may need decisional support [1]. All participants then received text-based education about AF, stroke risk in AF, and the need for anticoagulation. Following the education, we randomized patients to receive no visual (standard group), a visual representation of relevant probabilities of risk of stroke in AF (visual group), or to the new decision support tool that combined design-tailored visual displays with a values clarification (visual+VC group). The survey provider performed the randomization. Quotas were used to ensure adequate sex (50% female), race (maximum of 62.3% White), and ethnicity (minimum of 12.4% not Hispanic or Latino) across all groups. Randomization was done until those quotas were met, which led to more than 200 participants in each group.

**Figure 1.**
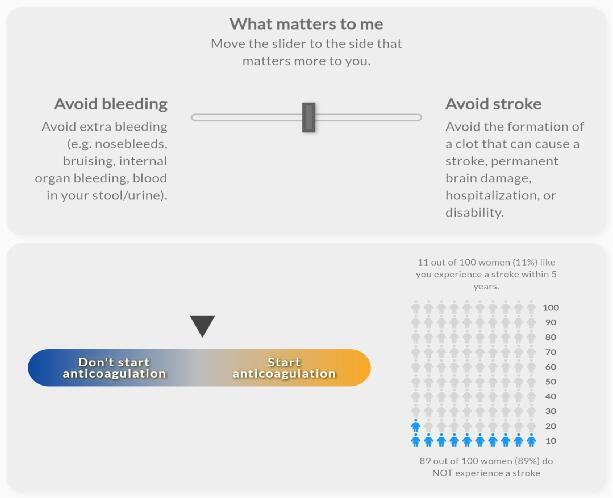
Example visualization of values clarification with icon array for a 75-year-old female with hypertension.

The values clarification group was presented with an exercise to evaluate which health event matters more to them: avoiding bleeding or stroke. This values clarification exercise altered the recommendation to “start anticoagulation” or “don’t start anticoagulation” based on a slider movement between the 2 health events. As the user moved the slider toward avoiding a stroke, the pointer moved toward the recommendation to “start anticoagulation.” As the user moved the slider toward avoiding bleeding, the pointer moved toward the recommendation to “don’t start anticoagulation.” In addition, those randomized to the visual or visual+VC group were subrandomized to receive either a gauge display showing the CHA_2_DS_2_-VASc score or an icon array representing the individual’s probability of experiencing a stroke using a person icon [7]. The individuals’ probability of experiencing a stroke did not change during the values clarification exercise. Figures1^4^ display examples of the 4 visualizations. Participants were also asked several questions to capture baseline characteristics. The complete survey, including consent, patient scenario, educational content, and questions, is available in Multimedia Appendix 1.

**Figure 2.**
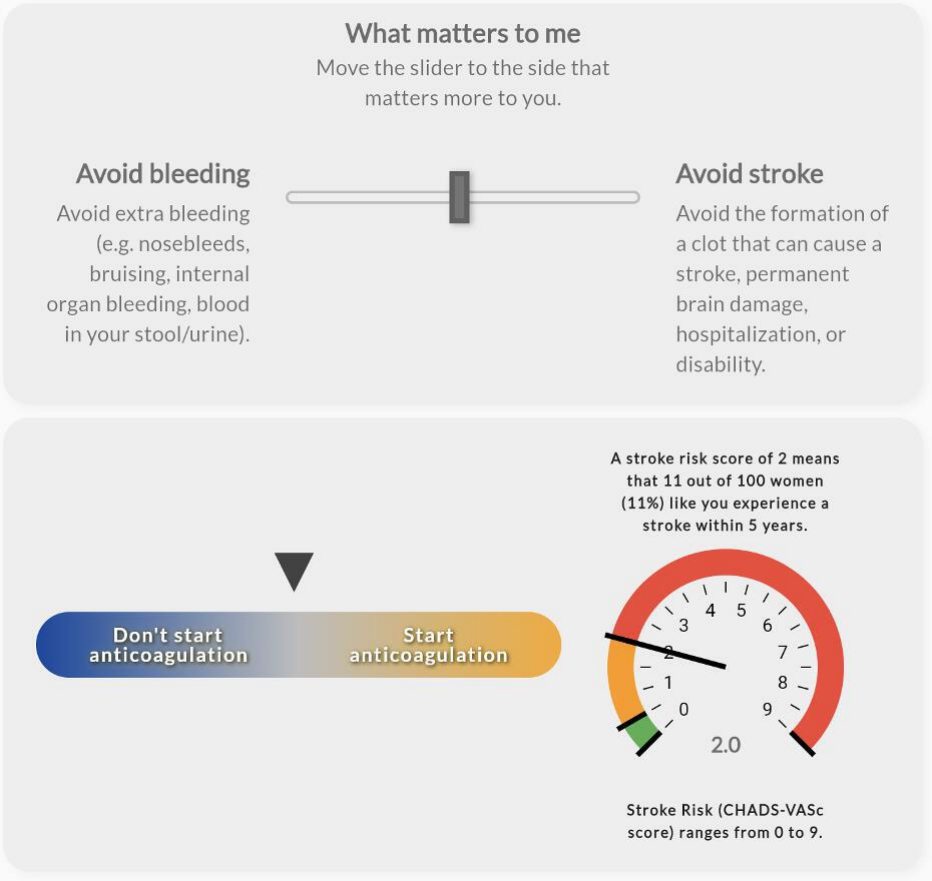
Example visualization of values clarification with gauge for a 75-year-old female with hypertension.

**Figure 3.**
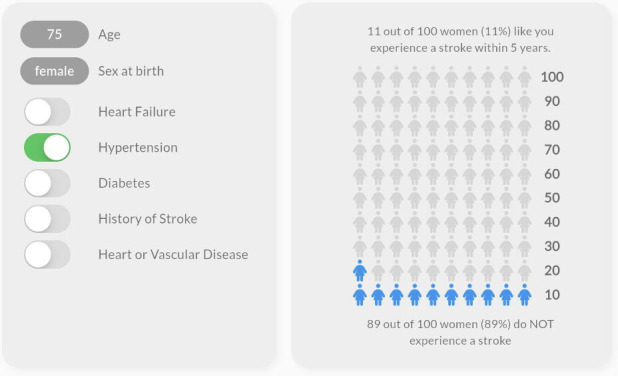
Example visualization with icon array for a 75-year-old female with hypertension.

**Figure 4.**
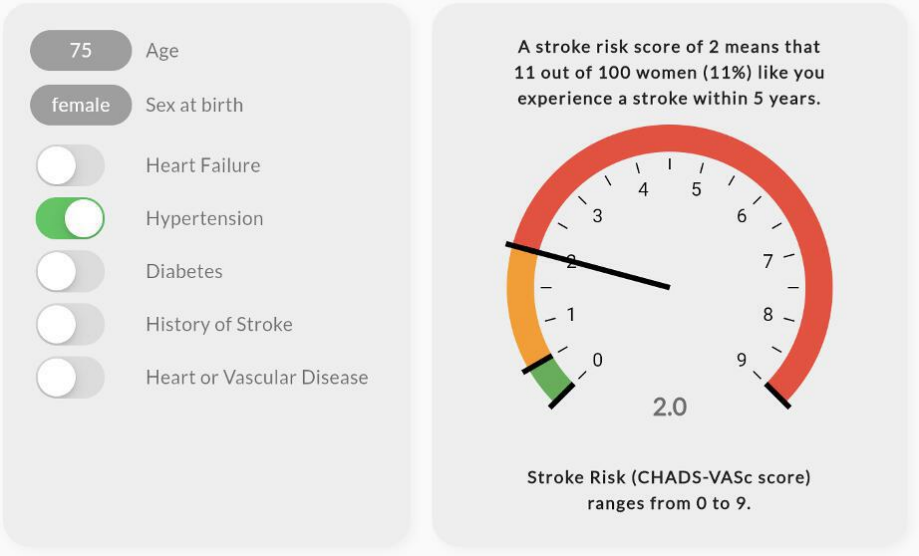
Example visualization with gauge for a 75-year-old female with hypertension.

### Outcomes

Participants completed the SURE (Sure of Myself; Understand Information; Risk-Benefit Ratio; Encouragement) screening test, which assesses the conflict a person has when making a decision [13]. The SURE test was used to understand if the participants in this study felt comfortable with their own decision to take or not take an OAC after reviewing the standard education or visuals. This was the primary outcome of this randomized trial [14]. The four yes-or-no questions are: (1) Do you feel SURE about the best choice for you? (2) Do you know the benefits and risks of each option? (3) Are you clear about which benefits and risks matter most to you? (4) Do you have enough support and advice to make a choice? Patient comfort was assessed as the percentage of participants answering yes to all the questions. Additionally, we measured anticoagulation intentions by the question: “Based on how you feel about this decision right now, would you say you will choose to,” with anchors, “Definitely TAKE an anticoagulant,” (100) on the right of the scale and, “Definitely NOT take an anticoagulant,” (0) on the left.

Secondary outcomes were questions about the participants’ understanding of anticoagulation for SPAF. The questions were: (1) How much of a reduction would anticoagulation make to your risk of stroke in AF? (0 to 100 scale: 0=Very small to 100=Very large); (2) How important is anticoagulation for SPAF? (0 to 100 scale: 0=Not at all important to 100=Very important); (3) How worried would you be about bleeding if you took anticoagulation for SPAF? (0 to 100 scale: Not at all worried to Very worried); and (4) How worried would you be about having a stroke if you did NOT take anticoagulation? (0 to 100 scale: Not at all worried to Very worried).

### Statistical Analysis

The study was powered to detect 10 percentage differences, for example, 50% of patients in the standard group versus 60% of patients in the visual group and 70% of patients in the visual+VC group answering “Yes” to all questions on the SURE test, the primary outcome. This was considered a clinically meaningful difference between experimental groups. A total sample size of 480 survey participants (160 in each group) provided greater than 90% power to detect such a difference using a chi-square test. We set our recruitment goal for this study at 200 participants in each arm to account for variation in the estimates. The SURE test was reported as a percent of participants answering “Yes” as the numerator and the total number of participants as the denominator. The secondary outcome questions were analyzed using an analysis of variance and reported as a mean and SD of the scale in each group.

### Ethical Considerations

This study was determined to be exempt by the University of Michigan Institutional Review Board (HUM00183776). Participants consented to participate in the survey study. Completed questionnaires were collected anonymously, and the data were deidentified. The service provider, Qualtrics, was paid for each participant that completed the survey. Compensation was provided by the service provider to the participants in the study.

## Results

### Baseline Characteristics

**Figure 5.**
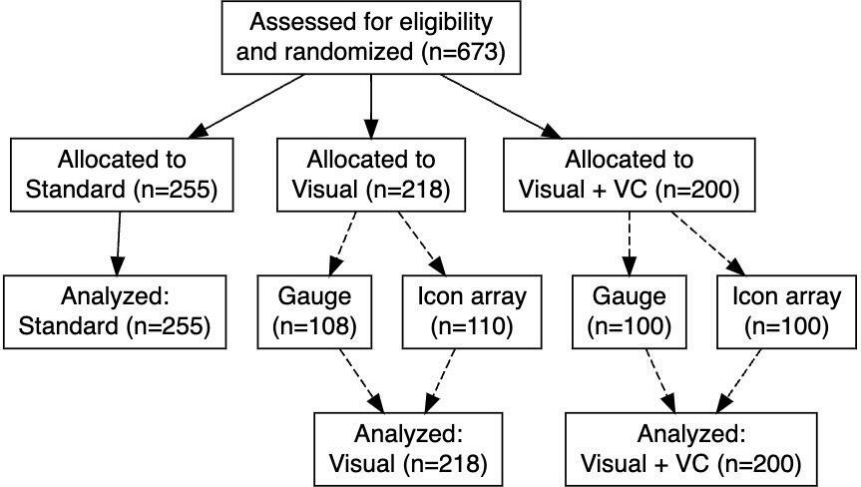
Flow diagram for patient enrollment, randomization, and analysis.

We recruited a total of 673 participants who completed the survey and were randomized to receive standard written communication (standard group), a visual representation of relevant probabilities (visual group), or the new decision support tool that combines design-tailored visual displays with values clarification (visual+VC group). Participant enrollment and allocation are summarized in the flow diagram (Figure 5). The average age was 54 (SD 6) years, and about half of the participants in the survey were female. Table 1 shows more detailed baseline demographics of the participants.

**Table 1.** Baseline characteristics.

Variable	Standard (n=255)	Visual (n=218)	Visual+VC (n=200)	*P* value
Age (years), mean (SD)	54.4 (5.8)	54.5 (5.8)	54.3 (6.1)	.93
Sex (female), n (%)	128 (50.2)	102 (46.8)	97 (48.5)	.76
Race, n (%)				.55
Black	34 (13.3)	27 (12.4)	26 (13)	
Other	29 (11.4)	21 (9.6)	24 (12)	
White	192 (75.3)	170 (78)	150 (75)	
Hispanic or Latino, n (%)	55 (21.5)	44 (20.2)	24 (12)	.02
Self-rated health status, n (%)				.68
Poor	4 (1.6)	8 (3.7)	7 (3.5)	
Fair	40 (15.7)	43 (19.7)	34 (17)	
Good	126 (49.4)	104 (47.7)	90 (45)	
Very good	66 (25.6)	51 (23.4)	57 (28.5)	
Excellent	19 (7.5)	12 (5.5)	12 (6)	
Seen an HCP^a^ in last 12 months, n (%)	196 (76.9)	162 (74.3)	156 (78)	.66
Prescription insurance, n (%)	210 (82.4)	177 (81.2)	164 (82)	.95
Knows someone with AFib^b^, n (%)	61 (23.9)	64 (29.4)	61 (30.5)	.23
Knows someone taking an OAC^c^, n (%)	115 (45.1)	103 (47.3)	103 (51.5)	.39
Confidence filling out forms, n (%)				.24
Never	6 (2.4)	3 (1.4)	1 (0.5)	
Occasionally	0 (0)	5 (2.3)	2 (1)	
Sometimes	18 (7.1)	11 (5.1)	10 (5)	
Often	42 (16.5)	39 (17.9)	40 (20)	
Always	189 (74.1)	160 (73.4)	147 (73.5)	
Help reading, n (%)	102 (40)	74 (33.9)	87 (43.5)	.13
Problems reading, n (%)	101 (39.6)	77 (35.2)	77 (38.5)	.62

a HCP: health care provider.

b AFib: atrial fibrillation.

c OAC: oral anticoagulation.

### SURE Test Results

The overall SURE test, saying “yes” to all 4 components, was 61.2% (156/255) for the standard group, 66.5% (145/218) for the visual group, and 67% (134/200) for the visual+VC group (visual vs standard, odds ratio [OR] 1.26, 95% CI 0.86‐1.84; *P*=.23; visual+VC vs standard, OR 1.29, 95% CI 0.87‐1.90; *P*=.20). In exploratory analyses of each question, participants felt more sure about the best choice for them, question 1 of the SURE test, if they were presented with either visual compared to standard education (visual vs standard, OR 1.59, 95% CI 1.01‐2.49; *P*=.04; visual+VC vs standard, OR 1.48, 95% CI 0.94‐2.33; *P*=.09). Table 2 shows the overall SURE test and the individual components.

**Table 2.** SURE^a^ test by group.

Variable	Standard, n (%)	Visual, n (%)	Visual+VC, n (%)	OR^b^ (95% CI) and *P* value
Yes to all 4 SURE questions	156 (61.2)	145 (66.5)	134 (67)	Visual versus No Visual: 1.26 (0.86‐1.84); *P*=.23 Visual+VC versus No Visual: 1.29 (0.87‐1.90); *P*=.20
Do you feel SURE about the best choice for you? Yes	191 (74.9)	180 (82.6)	163 (81.5)	Visual versus No Visual: 1.59 (1.01‐2.49); *P*=.04 Visual+VC versus No Visual: 1.48 (0.94‐2.33); *P*=.09
Do you know the benefits and risks of each option? Yes	224 (87.8)	193 (88.5)	179 (89.5)	Visual versus No Visual: 1.07 (0.61‐1.87); *P*=.82 Visual+VC versus No Visual 1.18 (0.66‐2.12); *P*=.59
Are you clear about which benefits and risks matter most to you? Yes	225 (88.2)	185 (84.9)	173 (86.5)	Visual versus No Visual: 0.75 (0.44‐1.27); *P*=.28 Visual+VC versus No Visual: 0.85 (0.49‐1.49); *P*=.58
Do you have enough support and advice to make a choice? Yes	189 (74.1)	167 (76.6)	151 (75.5)	Visual versus No Visual: 1.14 (0.75‐1.74); *P*=.53 Visual + VC versus No Visual: 1.08 (0.70‐1.65); *P*=.65

a SURE: Sure of Myself; Understand Information; Risk-Benefit Ratio; Encouragement.

b OR: odds ratio.

Participants were less likely to choose to take an OAC when shown either visual compared to standard education. The average rating was 58.3 (SD 30) in the standard group, 51.4 (SD 32) in the visual group, and 51.9 (SD 28) in the visual+VC group (*P*=.03). Participants also felt that the reduction in stroke risk from an OAC was less in either visual group than in the standard education group. The average rating was 63.8 (SD 22) in the standard group, 54.2 (SD 28) in the visual group, and 58.6 (SD 25) in the visual+VC group (*P*<.001). Table 3 demonstrates more detail on the questions about choosing OAC and stroke risk.

**Table 3.** Questions about choosing OAC^a^ and stroke risk by group.

Variable	Standard, mean (SD)	Visual, mean (SD)	Visual+VC, mean (SD)	*P* value
Based on how you feel about this decision right now, would you say you will choose to: 0=Do not take OAC, 100=Take OAC	58.3 (30.0)	51.4 (32.0)	51.9 (28.0)	.03
How much of a reduction would anticoagulation make to your risk of stroke in AFib^b^? 0=very small, 100=very large	63.8 (22.0)	54.2 (28.0)	58.6 (25.0)	<.001
How important is anticoagulation for stroke prevention in AFib? 0=Not important, 100=Extremely important	75.6 (18.0)	75.7 (19.0)	73.9 (16.0)	.55
How worried would you be about bleeding if you took anticoagulation for stroke prevention in AFib? 0=Not worried, 100=Extremely worried	64.3 (24.0)	65.2 (25.0)	63 (23.0)	.63
How worried would you be about having a stroke if you did NOT take anticoagulation? 0=Not worried, 100=Extremely worried	66.3 (26.0)	63 (28.0)	62.1 (26.0)	.21

a OAC: oral anticoagulation.

b AFib: atrial fibrillation.

No significant differences were found between the visualization methods, gauge, and icon array for the outcome of the SURE test. Participants answered “yes” to all 4 SURE test questions, 65.9% (137/208) when shown a gauge and 67.6% (142/210) when shown an icon array group (*P*=.70). Participants were less likely to choose to take an OAC when shown a gauge compared to an icon array (mean 48.8, SD 31 vs mean 55.4, SD 30; *P*=.03). Participants also felt that the reduction in stroke risk from an OAC was less when shown a gauge than an icon array (mean 52.1, SD 27 vs mean 60.4, SD 25; *P*=.001). Table 4 provides further details regarding choosing OAC and stroke risk by visualization method.

**Table 4.** Questions about choosing OAC^a^ and stroke risk by visualization method.

Variable	Gauge (n=208), mean (SD)	Icon array (n=210), mean (SD)	*P* value
Based on how you feel about this decision right now, would you say you will choose to: 0=Do not take OAC, 100=Take OAC	48.8 (31.0)	55.4 (30.0)	.03
How much of a reduction would anticoagulation make to your risk of stroke in AFib^b^? 0=very small, 100=very large	52.1 (27.0)	60.4 (25.0)	.001
How important is anticoagulation for stroke prevention in AFib? 0=Not important, 100=Extremely important	74.6 (17.0)	75.1 (18.0)	.76
How worried would you be about bleeding if you took anticoagulation for stroke prevention in AFib? 0=Not worried, 100=Extremely worried	64.5 (24.0)	63.7 (24.0)	.73
How worried would you be about having a stroke if you did NOT take anticoagulation? 0=Not worried, 100=Extremely worried	60.5 (27.0)	64.7 (27.0)	.11

a OAC: oral anticoagulation.

b AFib: atrial fibrillation.

## Discussion

### Principal Results

This trial investigated the difference in participant preferences for OAC for SPAF after reviewing 3 different approaches, which included standard education (standard group), a visual representation of relevant probabilities of risk of stroke in AF (visual group), or the new decision support tool that combined design-tailored visual displays with a values clarification (visual+VC group). The visuals were created using a user-centered design approach with iterative feedback from patients and providers. These visuals are unique because of the addition of values clarification and because most current tools use a dot-based icon array to show stroke risk in AF [15,16]. Each participant was given a scenario with a CHA_2_DS_2_-VASc risk score, and the guidelines do not expressly state whether a patient should be prescribed an OAC. The 3 strategies did not affect the participants’ comfort in deciding to take an OAC between study groups, measured by the SURE test.

Participants were less likely to take an OAC and felt that the reduction in stroke risk from an OAC was less when shown either the visual or visual VC compared to standard education. This is unique for the CHA_2_DS_2_-VASc score of 1 for men and 2 for women, which we showed participants. Since the guidelines do not recommend for or against OAC in this population, visuals like the ones in this study could persuade patients not to take OAC.

Interestingly, the values clarification visual did not demonstrate a difference in the participants’ comfort in taking an OAC compared to the other visual group. This could have been due to several factors. Based on patient feedback, we used a horizontal bar for the values clarification. Previous versions of the tool we created and those in the literature used a vertical bar to represent the values clarification [8]. The horizontal bar could have led to more confusion than vertical bars. Additionally, the participants in this study were older than those in other studies using values clarification. Older participants may need more in-person help with the visuals. This could have led to more confusion with the intent of the visuals.

Although not the study’s primary outcome, the 2 visual types, gauge or icon array, influenced the participants’ decision to take an OAC and changed their perception of the stroke risk reduction from an OAC compared to the person-based icon array. Showing risk with the gauge made participants less likely to take an OAC, and they felt that the reduction in stroke risk from an OAC was smaller than the icon array. A body of research demonstrates the value of icon arrays in risk communication [17-20]. This difference in risk demonstration in this study could be explained by the lower detail presented in the gauge compared to the icon array, which represents a matrix of icons showing the at-risk population. The more detailed icon array could have made it easier for participants to understand the estimated risk and decide to take an OAC.

### Limitations

There are several limitations to this study. First, the tool is meant for a shared decision-making session with a patient and provider, but the survey was done with members of the general public. Second, the survey was conducted with the general public to decrease any bias the provider would add to the shared decision-making situation in the study. If this tool was implemented as shared decision-making with a provider, it could lead to a better understanding of the tool. Future research should investigate the use of the tool with a provider present to guide and educate the patient. Third, newer AF guidelines have been published since the time of the study’s completion. Although our methods and educational materials referred to earlier guidelines, the updated guidelines recognize a borderline stroke-risk threshold (eg, CHA₂DS₂-VASc of 1 for men or 2 for women) where shared decision-making remains a priority.

### Conclusions

Overall, the study suggests visual aids can modestly affect decision confidence and perceptions regarding the benefits of anticoagulation therapy but do not significantly change overall decision certainty in a scenario where the guidelines do not recommend for or against the treatment. Future work should determine the role of a gauge versus icon array in visual aids for decision-making in SPAF.

## Supplementary material

10.2196/67956 Multimedia Appendix 1 Qualtrics Survey.

## Abbreviations

AF: atrial fibrillation
CHA_2_DS_2_-VASc: congestive heart failure; hypertension; age ≥75 years [doubled]; type 2 diabetes; previous stroke, transient ischemic attack, or thromboembolism [doubled]; vascular disease; age 65 to 75 years; and sex category
HAS-BLED: hypertension, abnormal renal/liver function, stroke, bleeding history or predisposition, labile international normalized ratio, elderly [>65 years], drugs/alcohol concomitantly
OAC: oral anticoagulation
ODSF: Ottawa Decision Support Framework
OR: odds ratio
SPAF: stroke prevention in atrial fibrillation
SURE: Sure of Myself; Understand Information; Risk-Benefit Ratio; Encouragement
visual+VC: visual aids plus a values clarification exercise

## References

[1] Joglar JA, Chung MK, Armbruster AL (2024). 2023 ACC/AHA/ACCP/HRS guideline for the diagnosis and management of atrial fibrillation: a report of the American College of Cardiology/American Heart Association Joint Committee on clinical practice guidelines. Circulation.

[2] Hart RG, Pearce LA, Aguilar MI (2007). Meta-analysis: antithrombotic therapy to prevent stroke in patients who have nonvalvular atrial fibrillation. Ann Intern Med.

[3] Noseworthy PA, Brito JP, Kunneman M (2019). Shared decision-making in atrial fibrillation: navigating complex issues in partnership with the patient. J Interv Card Electrophysiol.

[4] Lip GYH, Frison L, Halperin JL, Lane DA (2010). Identifying patients at high risk for stroke despite anticoagulation: a comparison of contemporary stroke risk stratification schemes in an anticoagulated atrial fibrillation cohort. Stroke.

[5] van den Ham HA, Klungel OH, Singer DE, Leufkens HGM, van Staa TP (2015). Comparative performance of ATRIA, CHADS2, and CHA2DS2-VASc risk scores predicting stroke in patients with atrial fibrillation: results from a National Primary Care Database. J Am Coll Cardiol.

[6] Pisters R, Lane DA, Nieuwlaat R, de Vos CB, Crijns H, Lip GYH (2010). A novel user-friendly score (HAS-BLED) to assess 1-year risk of major bleeding in patients with atrial fibrillation: the Euro Heart Survey. Chest.

[7] Zikmund-Fisher BJ, Witteman HO, Dickson M (2014). Blocks, ovals, or people? Icon type affects risk perceptions and recall of pictographs. Med Decis Making.

[8] Witteman HO, Chipenda Dansokho S, Exe N, Dupuis A, Provencher T, Zikmund-Fisher BJ (2015). Risk communication, values clarification, and vaccination decisions. Risk Anal.

[9] Witteman HO, Scherer LD, Gavaruzzi T (2016). Design features of explicit values clarification methods: a systematic review. Med Decis Making.

[10] Witteman HO, Gavaruzzi T, Scherer LD (2016). Effects of design features of explicit values clarification methods: a systematic review. Med Decis Making.

[11] Murray MA, Miller T, Fiset V, O’Connor A, Jacobsen MJ (2004). Decision support: helping patients and families to find a balance at the end of life. Int J Palliat Nurs.

[12] O’Connor AM, Tugwell P, Wells GA (1998). A decision aid for women considering hormone therapy after menopause: decision support framework and evaluation. Patient Educ Couns.

[13] Légaré F, Kearing S, Clay K (2010). Are you SURE?: Assessing patient decisional conflict with a 4-item screening test. Can Fam Physician.

[14] Parayre AF, Labrecque M, Rousseau M, Turcotte S, Légaré F (2014). Validation of SURE, a four-item clinical checklist for detecting decisional conflict in patients. Med Decis Making.

[15] Kunneman M, Branda ME, Hargraves IG (2020). Assessment of shared decision-making for stroke prevention in patients with atrial fibrillation: a randomized clinical trial. JAMA Intern Med.

[16] Noseworthy PA, Branda ME, Kunneman M (2022). Effect of shared decision-making for stroke prevention on treatment adherence and safety outcomes in patients with atrial fibrillation: a randomized clinical trial. J Am Heart Assoc.

[17] Hawley ST, Zikmund-Fisher B, Ubel P, Jancovic A, Lucas T, Fagerlin A (2008). The impact of the format of graphical presentation on health-related knowledge and treatment choices. Patient Educ Couns.

[18] Tait AR, Voepel-Lewis T, Zikmund-Fisher BJ, Fagerlin A (2010). The effect of format on parents’ understanding of the risks and benefits of clinical research: a comparison between text, tables, and graphics. J Health Commun.

[19] Galesic M, Garcia-Retamero R, Gigerenzer G (2009). Using icon arrays to communicate medical risks: overcoming low numeracy. Health Psychol.

[20] Garcia-Retamero R, Galesic M (2010). Who proficts from visual aids: overcoming challenges in people’s understanding of risks. Soc Sci Med.

