# Supplementary material for: A Web-Based Tool to Perform a Values Clarification for Stroke Prevention in Patients With Atrial Fibrillation: Design and Preliminary Testing Study
Source: JMIR Cardio. 2025 Apr 11;9:e67956. doi: 10.2196/67956 (PMC12007723; doi:10.2196/67956)
Supplement: Multimedia Appendix 1 [file cardio-v9-e67956-s001.pdf]

Consent

Information Sheet
Understanding the Risks and Benefits of Anticoagulation in AFib
HUM# 00183776

Principal Investigator: Michael Dorsch, PharmD, MS,
Assistant Professor of Clinical Pharmacy
University of Michigan

You are invited to participate in a research study about a heart condition called atrial fibrillation which is commonly referred to as AFib. The study team is investigating the best way to teach people about the risks and benefits of starting anticoagulation (blood thinners) as a treatment for AFib.

If you agree to be part of the research study, you will be asked to review information about the risks and benefits of starting anticoagulation as a treatment for AFib. After reviewing the materials, you will be asked to answer a series of questions about the risks and benefits of anticoagulation.

Benefits of the research: Participating in the interview may not benefit you directly. You could learn more about anticoagulation and atrial fibrillation. We hope what we learn from you will help other people in the future.

Risks and discomforts: There are no anticipated risks or discomforts with this survey. You may choose to end the survey at any time.

Compensation: You will be compensated for your time by your panel provider.

Participating in this study is completely voluntary. Even if you decide to participate now, you may change your mind and stop at any time.

No identifiable data will be collected in this study. Information collected in this project may be shared with other researchers.

If you have questions about this research study, please contact:
Michael Dorsch, PharmD, MS
dorsch-lab@umich.edu

The University of Michigan's Health Sciences and Behavioral Sciences Institutional Review Board has determined that this study is exempt from IRB oversight (HUM00183776).

Before we have you think about a medical scenario, we need to check that you are eligible for this survey.

Have you ever been diagnosed with atrial fibrillation (also known as AFib)?

- ○ Yes
- ○ No

Have you ever taken anticoagulants (also known as blood thinners)?

- ○ Yes
- ○ No

What is your age?

[ ]

Sex at birth

- ○ Male
- ○ Female

What is your race? Select all that apply.

- ☐ American Indian or Alaska Native
- ☐ Asian
- ☐ Black or African American

- [ ] Native Hawaiian or Other Pacific Islander
- [ ] White

What is your ethnicity?

- ○ Hispanic or Latino
- ○ Not Hispanic or Latino

Patient_scenario

Pretend that the following scenario is you. It is not real life, but focus on how you would react if this were true in your life.

You go in to see your doctor for an annual check-up and your doctor performs some standard tests.

Your doctor says, “Has anyone ever told you that you have an irregular heartbeat?”

You say, “No.”

Your doctor says, “I noticed on your tests that you have an irregular heartbeat, known as atrial fibrillation or AFib for short.

Since you have a history of high blood pressure or hypertension, there is a risk of stroke. When people have a risk of stroke from AFib, we discuss the best treatment to lower that stroke risk.

Some patients choose to take a pill known as a blood thinner or anticoagulant, some patients choose not to take that pill.

Since there is no right answer in your case, here is some more information to help you with the decision about whether to take an anticoagulant or not.”

Patient Education

The following is education about AFib:

Atrial fibrillation, also known as AFib, is an **irregular heartbeat** and is a very common heart problem as people get older.

The irregular heartbeats from AFib can cause blood to pool in the upper chambers of the heart. Blood pooling from AFib can lead to a **blood clot.** The blood clot may travel to the brain causing a **stroke**. A stroke can lead to permanent brain damage, hospitalization, or disability.

Just to confirm your understanding, AFib can lead to which of the following problem?

- ○ Stroke
- ○ High blood pressure
- ○ Diabetes
- ○ High cholesterol

The following is education about stroke risk with AFib:

Each person with AFib has a different risk of a stroke. The risk of stroke for AFib is based on your age, gender, and whether you have high blood pressure, diabetes, heart disease, or a history of stroke. As you get older you are at higher risk of stroke in AFib. Women are at higher risk of stroke in AFib. Patients who have high blood pressure, diabetes, certain heart diseases, or a history of stroke are at higher risk of stroke in AFib.

The following is education on the use of anticoagulation in AFib:

Anticoagulants, also known as blood-thinners, are used to prevent stroke in AFib. The risks and benefits of anticoagulants should be weighed by each person. Anticoagulants can cause nosebleeds, bruising, blood in your stool/urine, or internal organ bleeding. Some common anticoagulants include warfarin (Coumadin), apixaban (Eliquis), and rivaroxaban (Xarelto).

While anticoagulants lower the risk of stroke in AFib, they also increase the risk of bleeding.

Patients without risk factors of stroke in AFib are not prescribed an anticoagulant.

When a patient has 1 or 2 risk factors of stroke in AFib, they may or may not be prescribed an anticoagulant.

NoVisuals

ChoicesIconArrayOnly

This visual shows your stroke risk in AFib:

ChoicesGaugeOnly

This visual shows your stroke risk in AFib:

ResultsIconArray

This visual shows your stroke risk in AFib:

This visual clarifies your values about choosing to use an anticoagulant to reduce the risk of stroke in AFib:

ResultsGauge

This visual shows your stroke risk in AFib:

This visual clarifies your values about choosing to use an anticoagulant to reduce the risk of stroke in AFib:

Surveys

Having now learned about your stroke risk in AFib, please answer the following question.

Based on how you feel about this decision right now, would you say you will choose to:

Definitely do NOT take anticoagulant	Definitely TAKE anticoagulant

Based on your answer to the previous question and what you learned about stroke risk in AFib, the following questions are clarifying how sure you are with your decision.

Do you feel SURE about the best choice for you?

- ◯ Yes
- ◯ No

Do you know the benefits and risks of each option?

- ◯ Yes
- ◯ No

Are you clear about which benefits and risks matter most to you?

- ◯ Yes
- ◯ No

Do you have enough support and advice to make a choice?

- ◯ Yes
- ◯ No

Based on your scenario and what you learned about stroke prevention in AFib, please answer the following questions.

How much of a reduction would anticoagulation make to your risk of stroke in AFib?

Very small | Very large

How important is anticoagulation for stroke prevention in AFib?

Not important at all | Extremely important

How worried would you be about bleeding if you took anticoagulation for stroke prevention in AFib?

Not worried at all | Extremely worried

How worried would you be about having a stroke if you did NOT take anticoagulation?

Not worried at all | Extremely worried

How often do you have someone help you read hospital materials (like a family member, friend, hospital/ clinic worker or caregiver)?

- ○ All of the time
- ○ Most of the time
- ○ Some of the time
- ○ A little of the time
- ○ None of the time

How often do you have problems learning about your medical condition because of difficulty understanding written information?

- ◯ All of the time
- ◯ Most of the time
- ◯ Some of the time
- ◯ A little of the time
- ◯ None of the time

How confident are you filling out forms by yourself?

- ◯ Always
- ◯ Often
- ◯ Sometimes
- ◯ Occasionally
- ◯ Never

For each of the following questions, please check the box that best reflects how good you are at doing the following things:

How good are you at working with fractions?

Not at all good 1	2	3	4	5	Extremely good 6
◯	◯	◯	◯	◯	◯

How good are you at working with percentages?

Not at all good 1	2	3	4	5	Extremely good 6
◯	◯	◯	◯	◯	◯

How good are you at calculating a 15% tip?

Not at all good 1	2	3	4	5	Extremely good 6
◯	◯	◯	◯	◯	◯

How good are you at figuring out how much a shirt will cost if it is 25% off?

Not at all good 1	2	3	4	5	Extremely good 6
○	○	○	○	○	○

For each of the following questions, please check the box that best reflects your answer:

When reading the newspaper, how helpful do you find tables and graphs that are parts of a story?

Not at all helpful 1	2	3	4	5	Extremely helpful 6
○	○	○	○	○	○

When people tell you the chance of something happening, do you prefer that they use words ("it rarely happens") or numbers ("there's a 1% chance")?

Always prefer words 1	2	3	4	5	Always prefer numbers 6
○	○	○	○	○	○

When you hear a weather forecast, do you prefer predictions using percentages (e.g., “there will be a 20% chance of rain today”) or predictions using only words (e.g., “there is a small chance of rain today”)?

Always prefer percentages 1	2	3	4	5	Always prefer words 6
○	○	○	○	○	○

How often do you find numerical information to be useful?

Never 1	2	3	4	5	Very often 6
○	○	○	○	○	○

Sometimes medical action is clearly necessary, and sometimes it is clearly NOT necessary. Other times, reasonable people differ in their beliefs about whether medical action is needed.

In situations where it's NOT clear, do you tend to lean towards **taking action** or do you lean towards **waiting and seeing** if action is needed?

Importantly, there is no "right" way to be.

I strongly lean towards waiting and seeing.	I lean towards waiting and seeing.	I somewhat lean towards waiting and seeing.	I somewhat lean towards taking action.	I lean towards taking action.	I strongly lean towards taking action.
○	○	○	○	○	○

When making decisions about medical care, do you tend to lean towards doing **only what is necessary** or do you lean towards doing **everything possible**?

I strongly lean towards doing only what is necessary.	I lean towards doing only what is necessary.	I somewhat lean towards doing only what is necessary.	I somewhat lean towards doing everything possible.	I lean towards doing everything possible.	I strongly lean towards doing everything possible.
○	○	○	○	○	○

Now we are going to ask you some questions about yourself.

What is the highest degree or level of school you have completed?

- ○ No schooling completed
- ○ Nursery school to 8th grade
- ○ Some high school, no diploma
- ○ High school graduate, diploma or the equivalent (for example: GED)
- ○ Some college credit, no degree
- ○ Trade/technical/vocational training
- ○ Associate degree
- ○ Bachelor's degree
- ○ Master's degree
- ○ Doctorate degree

Would you say your health is:

- ◯ Poor
- ◯ Fair
- ◯ Good
- ◯ Very good
- ◯ Excellent

From the list below, please select any of the health-related items that apply to you.

- ☐ Diagnosed with any heart condition
- ☐ Diagnosed with a chronic medical condition
- ☐ Had discussions with a healthcare provider about blood thinners
- ☐ None of the above

Have you seen a healthcare professional in the past year? This includes in-person, video or telephone visits.

- ◯ Yes
- ◯ No

Do you have prescription drug insurance?

- ◯ Yes
- ◯ No

Do you know anyone who has been diagnosed with AFib?

- ◯ Yes
- ◯ No

Do you know anyone that has taken an anticoagulant (blood thinner) medication?

- ◯ Yes
- ◯ No

What is your total household income?

- Less than $10,000
- $10,000 to $19,999
- $20,000 to $29,999
- $30,000 to $39,999
- $40,000 to $49,999
- $50,000 to $59,999
- $60,000 to $69,999
- $70,000 to $79,999
- $80,000 to $89,999
- $90,000 to $99,999
- $100,000 to $149,999
- $150,000 or more

Powered by Qualtrics
